# Exploring the movement dynamics of deception

**DOI:** 10.3389/fpsyg.2013.00140

**Published:** 2013-03-27

**Authors:** Nicholas D. Duran, Rick Dale, Christopher T. Kello, Chris N. H. Street, Daniel C. Richardson

**Affiliations:** ^1^Cognitive and Information Sciences, University of California MercedMerced, CA, USA; ^2^Cognitive, Perceptual and Brain Sciences, University College LondonLondon, UK

**Keywords:** deception, non-linear measures, Dynamical Systems Theory, embodiment, recurrence quantification analysis, multiscale entropy analysis, body and facial movements, time series analysis

## Abstract

Both the science and the everyday practice of detecting a lie rest on the same assumption: hidden cognitive states that the liar would like to remain hidden nevertheless influence observable behavior. This assumption has good evidence. The insights of professional interrogators, anecdotal evidence, and body language textbooks have all built up a sizeable catalog of non-verbal cues that have been claimed to distinguish deceptive and truthful behavior. Typically, these cues are discrete, individual behaviors—a hand touching a mouth, the rise of a brow—that distinguish lies from truths solely in terms of their frequency or duration. Research to date has failed to establish any of these non-verbal cues as a reliable marker of deception. Here we argue that perhaps this is because simple tallies of behavior can miss out on the rich but subtle organization of behavior as it unfolds over time. Research in cognitive science from a dynamical systems perspective has shown that behavior is structured across multiple timescales, with more or less regularity and structure. Using tools that are sensitive to these dynamics, we analyzed body motion data from an experiment that put participants in a realistic situation of choosing, or not, to lie to an experimenter. Our analyses indicate that when being deceptive, continuous fluctuations of movement in the upper face, and somewhat in the arms, are characterized by dynamical properties of less stability, but greater complexity. For the upper face, these distinctions are present despite no apparent differences in the overall amount of movement between deception and truth. We suggest that these unique dynamical signatures of motion are indicative of both the cognitive demands inherent to deception and the need to respond adaptively in a social context.

## Introduction

The keystone of “dynamical cognition” is the intimate relationship between mental and motor processes. Rather than the mind being limited to abstract computation, encapsulated from the body and its interactions with the environment, the connections between cognition, action, and perception are tightly intertwined (Port and Van Gelder, 1995; Riley et al., 2012). Consider the interlocked rhythms of speech and gesture, where hand and arm movements are timed to coincide with the articulation of words and phrases during communication. The exact timings suggest that information carried in gesture subserves the transmission of meaning, with both arising from the same underlying cognitive processes (McNeill, 1996). Such a relationship counters notions that the path between cognition and movement is one of discrete, sequential steps, where instructions to act are handed down from a central executive. Instead, cognition and action formed a coupled system that co-varies in systematic ways.

The connection between thought and action also suggests that hidden cognitive processes can be revealed in the dynamics of movement, such as those that occur during deception. Indeed, deception likely elicits unique cognitive demands that vary markedly from truthful communication (Vrij et al., 2010). By definition, deception requires mental partitioning of what is and what is not the case, and an intentional effort to convince listeners of the latter. In addition, it often occurs face-to-face, where a large array of motor cues are available, from movements of the hands and eyes, to facial movements and changes in articulatory patterns. Given this mind–body relationship, the possible consequences on deceptive behavior have not gone unstudied. However, overwhelming focus has been placed on discrete individual behaviors that can be noted and counted by human observers (e.g., see Vrij et al., 1996; Hill and Craig, 2002). In doing so, the dynamics of how movements are patterned across time have not been examined, and may in part explain why detection reliability in existing studies remains quite low (Bond and DePaulo, 2006).

Here, we take a different tack by examining the moment-by-moment temporal dependencies that reside in patterns of motion. At this more granular level, we are able to provide a *dynamical systems* account of deceivers' continuous movements in naturalistic contexts. By examining how fluctuations of movement are structured in time, new insights can be had about the manner in which mental dynamics are expressed in bodily dynamics. These insights are particularly relevant for evaluating existing studies based on an implicit assumption that deception negatively interferes with normal processes of communication. Such an assumption leads to explanations that are typically couched in terms of greater processing load, whereby attentional resources are presumably diverted away from, or overly committed to, the control of action (Ekman and Friesen, 1972; DePaulo, 1992; DePaulo and Friedman, 1998; Vrij et al., 2008). A consequence is that normal behavior is believed to be impaired in some way, often evidenced by decreases in movement frequency and duration (DePaulo et al., 2003; Porter and ten Brinke, 2010; Vrij et al., 2010).

From a dynamical systems perspective, this conclusion is based on a relatively coarse relationship between mind and body. As will be discussed further in the following section (“Complexity in Movement Variability”), increases or decreases in movement can serve only as gross indicators of how the cognitive and motor systems are indeed impaired. Rather, what is most telling are the structural properties of *stability* and *complexity* that are derived from the fine-grained changes in movement variability. It is here that the influences of deception might be more directly revealed. We hypothesize that the outcome may not be one of impairment, but instead a reorganization of behavior over time that is better able to flexibly respond to the changing demands in deceptive contexts. Although we provide additional justification for this claim (see section “Adaptive Responding During Deception”), it is important to note that our arguments can only be, at present, speculative. Nonetheless, combining existing cognitive accounts of deception and deception detection with further exploration of dynamics may be a fruitful avenue of investigation. We will argue that dynamics may hold great promise in distinguishing deception from truth, as well as in understanding the underlying cognitive processes during deception.

We examine such possibilities by reanalyzing the bodily dynamics of participants in a deception experiment performed by Eapen et al. (2010). They designed two scenarios to elicit deception in participants who believed they were taking part in a study of mathematical ability and balance. Throughout the experiment, 29 points on the body, head, and on the face were rapidly sampled in three-dimensional space every 5 ms^1^.

In the first scenario, participants performed two math tests, and were offered a £5 reward if they performed better on the second test. Crucially, only they knew how well they actually performed on the second test, but since the difficulty was calibrated carefully, we could be confident that they performed worse.

As part of the second scenario, participants witnessed a laptop being accidently dropped by a junior investigator. In fact, the accident was staged and purposefully occurred while the senior researcher was out of the room. Later, the senior researcher returned, found his laptop not working, and asked the participant if anything had happened to it. Part of the participants' motivation to lie was the demeanor of the experimenters. The senior researcher was brusque and unpleasant throughout, but the junior researcher was very friendly toward the participant and expressed anxiety that she would be found out.

In both scenarios the participant was given the means, motive, and the opportunity to spontaneously lie to the experimenter. About 60% did so in each case. Eapen et al. found that while lying, compared to telling the truth, participants tended to move less. This conclusion was based on overall movement displacement across all motion points on the body. It echoes previous findings in the literature, albeit with a more refined, automated analysis. Here, we aim to extend these findings in two critical ways. First, by introducing two non-linear measures used in the biological and physical sciences that provide a novel analysis of the motor dynamics of deception. Second, by considering the theoretical implications that such characterizations of behavior have on the responsiveness of the cognitive system during deception. To better serve these goals, we turn next to an area of dynamical systems research that strongly motivates the current approach.

## Unraveling the dynamics of movement

### Complexity in movement variability

Even with the most basic types of control, the motor system faces the problem of how to constrain multiple and redundant bodily degrees of freedom in producing coherent, functional behaviors (Bernstein, 1967; Dickinson et al., 2000; Turvey, 2007). Given the countless physiological, contextual, and environmental interactions that are undoubtedly at play, assemblies of behavior cannot be captured by simple linear measures of more or less movement (Newell, 1998; Harbourne and Stergiou, 2009; Riley et al., 2012). Rather, the interactions are expressed as a process of self-organization, whereby the coordination of the musculoskeletal and nervous systems, coupled with ever-changing environmental demands, lead behavioral repertoires into stable response modes. To be maximally adaptive, movements should not stay fixed in any one mode, but must be able to rapidly transition to new stable modes of organization (Kelso, 1995; Port and Van Gelder, 1995; Riley and Turvey, 2002; Van Orden et al., 2003; Halley and Winkler, 2008). These transitions are the hallmark of complexity, expressed as short- and long-term dependencies in movement stability and instability.

The complexity exhibited in motor control also sheds new light on the influences of cognitive demand during processing tasks, an issue that is pertinent to deception. Despite the paucity of examples that can be drawn from the deception literature, this is offset by the extensive research involving the self-organization of postural control under dual-task conditions. The dual-task context is similar in form to deception, where one is trying to balance both what is true and what is a lie. In these postural dual-task designs, intentions and cognitive demands act to shape behavior in meaningful, albeit subtle ways. In a typical set-up, participants attempt to maintain an upright stance while performing cognitive tasks presented visually or auditorily, and that can vary in attentional and processing demands. The resulting outcomes suggest that there is no one-to-one correspondence between the cognitive constraints and how movements are expressed, such as saying that increased task difficulty leads to degraded movements (Riley et al., 2005; Fraizer and Mitra, 2008). Even when attentional resources are heavily drawn upon, the behavioral system does not necessarily break down, as would be the case if cognitive and motor processes were separate components competing for a limited pool of resources (e.g., as proposed in *limited capacity* theories, see Woollacott and Shumway-Cook, 2002; Schmidt, 2003; Schmidt and Lee, 2005, for review). Rather, because these cognitive and motor processes are tightly coupled, new solutions as to how to optimally redistribute resources are more quickly realized and expressed. Put simply, the cognitive system is not just breaking down or being overwhelmed, but is *reorganizing dynamically* in response to a new situation. How this might be relevant for deception in considered next.

### Adaptive responding during deception

Deception makes heavy demands on cognitive resources (see Vrij et al., 2011 for discussion). The truth also seems to be spontaneously activated with a lie, requiring additional effort to overcome (Osman et al., 2009; Duran et al., 2010). It is thought that performing concurrent tasks with deception, such as controlling one's body movements, will leave fewer resources available for successful deceptive performances (Leal et al., 2008). With less to work with, the movements of deceivers will become impaired in some way, whether it is an overall decrease in animation or overly controlled movements that appear rigid and unnatural (Zuckerman et al., 1981; Vrij et al., 1996; DePaulo and Friedman, 1998). However, from a dynamical systems perspective, this impairment interpretation does not necessarily reflect how the cognitive and motor systems are actually operating. Instead, the contextually and socially rich environment in which deception occurs provides a myriad of constraints that allow for the adaptive and functional reorganization of movement.

This view is inspired by Interpersonal Deception Theory (IDT), in which emphasis is placed on deceivers' ability to adapt within real-time interaction (Buller and Burgoon, 1996; Burgoon, 2005; Burgoon and Qin, 2006). Here, intentional and motivational factors allow deceivers to better regulate their behavior, doing so in a way that is highly responsive to their communication partner. According to this account, and the account considered here, deceptive displays of movement may not be driven by limited cognitive resources *per se* (i.e., impairment), but by the larger context. There is an important caveat however, in that IDT claims that resulting movements are largely under strategic control. We remain agnostic on this conclusion. Rather, our focus is on the reorganization of underlying “micro-behaviors” that are not intentionally controlled, and that may suggest a more subtle level of adaptivity. These movements are a non-conscious consequence of being on the ready in a situation that requires quick thinking and responsiveness in averting suspicion or detection. Finding greater complexity in the deceptive movements would support such a claim. Of course, if deceptive behavior has less complexity than honest behavior, doubt would be cast on our hypothesis and support would be lent to the impairment position. By adopting a dynamical systems approach, we can test these predictions.

We employed two measures used in the motor control literature, as well as the cognitive sciences more broadly. These two measures, recurrence quantification analysis (RQA) and multiscale entropy analysis (MSE), provide complementary insights into the structure (as opposed to the amount) of variability exhibited in motor behavior. They do so by quantifying patterns of stability and complexity of body movement, expressed as time series of marker positions in a motion capture system. In the sections that follow, we first turn to a more detailed, albeit introductory, tutorial of the conceptual and technical underpinnings of RQA and MSE (section “Quantifying the Structure in Time”). In the section “Extending an Analysis of Spontaneous Deception,” we outline the methodology from Eapen et al. (2010), and detail our analytical approach for reinterpreting the collected data, targeting the undifferentiated movements of the arms, head, and upper face. To draw distinctions between deceptive and truthful behavior, we then contrast a displacement measure of movement (a traditional summary approach) with the RQA and MSE results (section “Results and Interpretation”). Finally, we return to the theoretical and diagnostic potential of the current research in the discussion (section “Discussion”).

## Quantifying the structure in time

Human cognition is driven by many factors, all of which must work together in a coherent, integrated fashion. This multiscale characteristic is a hallmark of a complex, dynamical system. In such systems, subtle fluctuations of behavior may reveal transitions between stable behaviors, strategies, or states. If a system transitions frequently, this may reflect the buildup and breakdown of constraints over system elements as new potentials for movement are formed. Sticking to a single strategy will work against an individual when vigilance is required. These frequent transitions between strategies or states, then, maximize the potential for adaptive responding. To capture this underlying stability and complexity, a number of non-linear measures have been developed to quantify these properties (Seely and Macklem, 2004; Dale et al., 2011).

The first of the two measures employed here, RQA, makes use of a method called “phase-space reconstruction” to capture geometric properties of how a system evolves in time (Eckmann et al., 1987; Webber and Zbilut, 1994; Marwan et al., 2007). As will be explained below, a measure of stability can be derived based on how often a system revisits various regions within its phase space. In essence, more visits to the same region of phase space represents greater stability. The second measure, MSE, provides an assessment of system complexity as variation in sequences of observations in a time series, measured across different temporal window sizes (Costa et al., 2005; Gao et al., 2007). Rather than phase-space reconstruction, this measure is based on *sample entropy*, which is computed over coarse-grained versions of the original series. The result offers insights into meaningful complexity, where less complexity is a system with too few or excessive transitions across stable states, and is either locked into a limited number of behavioral repertoires, or devolves into stochastic noise. An example of a system with less complexity can be seen in the movements of young children who are first learning to walk (Newell, 1998). Their movements are often rigidly fixed or seemingly random, both conditions that suggest a lack of motor control in adapting to changing situational demands. Taken together, RQA and MSE may serve as powerful new tools for assessing non-linear changes in movement. In the next section, we flesh out the details of these methods in simple, qualitative terms^2^.

### Recurrence quantification analysis

As already touched upon, the idea of phase space is critical to RQA. It is worth carefully explaining the concept of a “phase space,” and how it is reconstructed from a time series. A phase space is defined by the variables (i.e., dimensions) that govern a dynamical system. For example, velocity and angle of the arms are necessary variables in explaining movement coordination, just as temperature and pressure are necessary variables for defining a thermodynamic system. Because these variables are time varying and directional, temporal succession over them produces a “behavioral trajectory” in a system's phase space. By examining the shape of the trajectory, it is possible to identify dynamic stabilities and instabilities as they emerge. One problem with this approach is that many state variables are unknown or cannot be measured. Another problem is the need to perform complex mathematics over a set of differential equations (e.g., integrating velocity vectors associated with state variables). To compensate, a solution is to reconstruct a phase space from time-lagged copies of a single time series of behavioral change. As originally observed by Takens (1981), a single state variable will be tightly coupled with all other state variables and thus is able to “stand in” for those that are unknown (Marwan, 2003; Stephen et al., 2009). Once plotted in high dimensional space, these surrogate variables are able to estimate the topography of system organization. Put simply, by analyzing just one behavioral time series, we can “reconstruct” the phase space.

**Figure 1:**
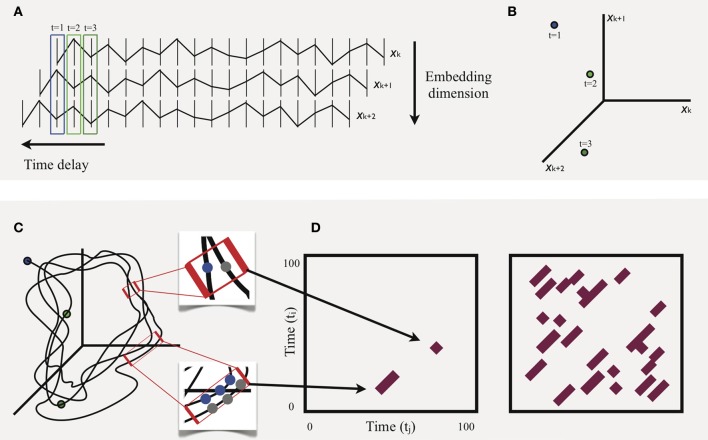
**Schematic illustration of the basic procedure of recurrence quantification analysis using a hypothetical example**.

Figure 1 provides an illustrative example of phase space reconstruction, as well as how RQA makes use of this space to derive measures that describe a system's behavior. To begin, in **(A)**, a univariate time series of movement fluctuation, *x*_*k*_, is shifted by any number of time steps (horizontal bars) to produce new *time-delayed* copies, *x*_*k* + 1_ and *x*_*k* + 2_, of the original series. The number of copies (i.e., *embedding dimensions*) is inferred to be the number of dimensions in which the system is really operating. These are limited to three for current purposes. The resulting vectors are then plotted in temporal order, with the first three time points, enclosed in colored boxes, plotted in **(B)**, and with all hypothetical points plotted in **(C)**. The result is a phase space trajectory that, from visual inspection, tends to pass through regions previously visited at earlier points in time. It is the proximity of these recurrent points that is crucial to RQA. Recurrent points, particularly sequences of recurrent points, indicate that the system is in a preferred region of its state space, i.e., an attractor. In the top inset of **(C)**, the Euclidean distance between two points, say at *t*_*i*_ = 45 and *t*_*j*_ = 85, fall within a predetermined *threshold radius* that defines a narrow region of space. When this occurs, it is simply plotted in what is known as a *recurrence plot*, shown in (**D**; left panel). Using the same logic, sequences of points that fall within the threshold radius are also captured: bottom inset of **(C)**. Thus, the corresponding diagonal in (**D**; left panel) can be interpreted as follows: the system at time points; *t*_*j*_ = 49, *t*_*j*_ = 50, *t*_*j*_ = 51, is also where the system was at points; *t*_*i*_ = 22, *t*_*i*_ = 23, *t*_*i*_ = 24; a stable region.

A complete (albeit hypothetical) recurrence plot is shown in (**D**; right panel). Properties of this plot provide the basis for all RQA measures. Here, we focus on just two: *percent recurrence* and *determinism*. The first is simply the percentage of filled points given the number of possible points, calculated according to the equation^3^,

$$RR=\frac{1}{N^{2}}\sum_{i,j=1}^{N}R_{i,j},$$

that counts all points between the two time series, (*i*, *j*), that fall within a predetermined radius. The latter, determinism, is the percentage of points that fall on diagonal lines, where diagonal lines indicate continuous sequences of repeating movements at different time points^4^. This is computed as a ratio between diagonal sequences and overall recurrence,

$$DET=\frac{\sum_{l=l_{\min}}^{N}lP(l)}{\sum_{i,j}^{N}R_{i,j}},$$

where *P*(*l*) = {*l*_*i*_; *i* = 1,…, *N*_*l*_} is the frequency distribution of all lengths of diagonal lines. Determinism is thus derived from basic recurrence, and is especially relevant for the current study. Specifically, it provides an intuitive measure of overall movement stability. However, as discussed earlier, determinism does not necessarily have a straightforward correspondence with system complexity. Movements that are highly predictable, occurring at regular, unchanging intervals, will exhibit high determinism, but are not complex. Likewise, movements characterized by random noise will show low determinism, but again are void of meaningful complexity. To identify what is meaningful, a suite of entropy-based measures has been developed that are based on the degree of repetitiveness in a time series. One measure in particular, MSE, provides a powerful technique for assessing complexity over multiple spatiotemporal scales in a single series, a method we turn to next^5^.

### Multiscale entropy

**Figure 2:**
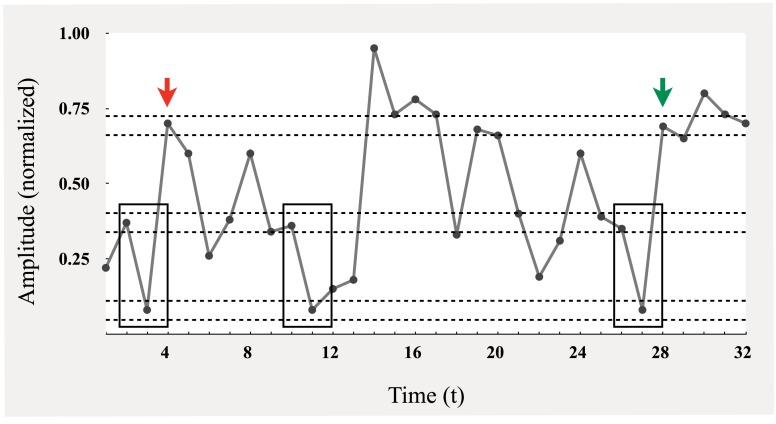
**Schematic illustration of the procedure for computing sample entropy [adapted from Costa et al. (2005)]**.

MSE is a two-step process, with the first step being the computation of sample entropy over a univariate time series. As previously stated, sample entropy is a measure of regularity, and captures, as Richman and Moorman (2000) observe, “the rate generation of new information.” This new information is related to the degree to which sequences of some length (*m*) in a time series remain similar after the sequence length is extended by an additional time point (*m* + 1). Figure 2, adapted from Costa et al. (2005), is presented to help conceptually ground what is meant by the given definition. A relevant pattern constitutes a short sequence of consecutive points, represented here as sequences of two points. This pattern is tallied as it repeats in the time series. For example, the consecutive values at *t* = 2 and *t* = 3 are a candidate pattern of interest (enclosed by box), and can be seen to repeat starting at *t* = 10 and at *t* = 27, as they occur within a similar range (or *threshold radius*; designated by horizontal dashed lines). This brings the total tally count to three. What needs to be determined is whether these two-point sequences can be extended by a similar, consecutive point. Returning to the original pattern in Figure 2, this value corresponds to *t* = 5 (marked by red arrow), and is only extendable at the *t* = 28 location (marked by green arrow), resulting in a tally of two three-point sequences. After repeating this process over all possible patterns, the natural log of the ratio between the final two-point and three-point tallies is computed. The result is sample entropy (a conditional probability), where greater values indicate that there are more two-point sequence patterns that cannot be extended by a similar third point; thus, there are a greater number of unique patterns, i.e., more information, greater complexity, and less regularity.

Although not immediately obvious, this measure has a fundamental problem in that higher entropy values also scale with increasing amounts of random noise (Costa et al., 2005). In other words, if there is less repetitiveness in a signal, it may not necessarily be due to complexity. One way to solve this problem is to evaluate how sample entropy changes over various spatiotemporal scales of the time series. Motor behavior is composed of a number of interacting elements that must come together to perform a task. Although these elements are closely bound and depend on each other for expression, each has its own intrinsic frequency that, when combined, produce organized structure across multiple spatiotemporal scales. The reader may ask: “What elements, what scales?” The relevant ones could be the various structures (head, torso, arms, etc.), cognitive processes (e.g., memory, language, etc.), and even finer-grained scales of neural organization. It is obvious that any organized cognitive performance, such as deception, is grounded in such an array of elements and processes. Yet, even without making any commitments about the physical or cognitive constraints on the system, this coherent self-organization is a fundamental characteristic of a dynamical process (Bar-Yam, 2004). Thus, a complex system reveals new information (complexity) across scales of decreasing frequency, whereas a random signal (void of underlying element interactions) will show less and less new information.

To produce a range of scales, the second step of MSE, the original time series is divided into non-overlapping windows of increasing sizes (i.e., coarse-graining). The values in each window are then averaged and replotted as a new point in a reduced series, producing a new time series, calculated by the following equation

$$y_{j}^{\left(\tau \right)}=1/\tau \sum_{i\;=(j\;-1)\tau +1}^{j\tau }x_{i},\;1\leq j\leq N/\tau .$$

**Figure 3:**
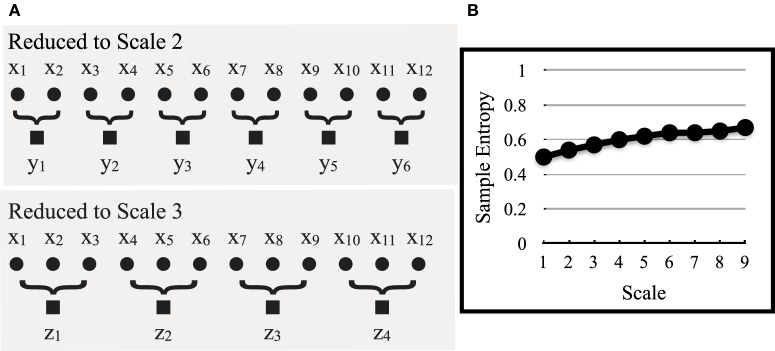
**In (A), the original time series, x_1–12_ (scale 1), is reduced by a lower-order scale to produce new time series, y_1–6_ (scale 2) and z_1–4_ (scale 3).** Although not shown, this continues to scale 9. In **(B)**, sample entropy is computed for these new lower frequency time series and plotted as a function of scale, from 1 to 9 [adapted from Costa et al. (2005)].

Here, the original time series, *X*_1_, …, *X*_*N*_, is divided into non-overlapping windows of length τ, with the datapoints in each window averaged to produce *y*^(τ)^_*j*_. An example of this process is shown in Figure 3 with an original time series of *x*_1_,…,*x*_12_ that is reduced by a scale of 2 (τ = 2), to *y*_1_, …, *y*_6_, and then by a scale of 3 (τ = 3), to *z*_1_, …, *z*_4_. In actual time series, which are comprised of thousands of points, reduction continues to a scale of 9 (τ = 9). These resulting scales correspond to signals of lower and lower frequencies. Finally, sample entropy is computed for each new reduced series and plotted with scale increasing along the x-axis (Figure 3B). The resulting curves are then used to compare relative differences between groups, an issue we return to when comparing deceptive and truthful movements in the following section.

## Extending an analysis of spontaneous deception

### Overview of Eapen et al. (2010)

To apply these dynamical techniques to deception, data captured during an interaction between a participant and two experimenters are explored here^6^. To ensure recordings were of natural spontaneous behavior, participants were told their behaviors would be captured while they took part in a study supposedly examining the relationship between mathematical ability and body sway. In reality, two critical recording periods were captured when the experiment was apparently at an end: one regarding their performance on a math test and the other regarding an accident they witnessed.

An amiable female experimenter welcomed participants. Soon after, a male experimenter entered and acted in a cold and unpleasant manner^7^. The male experimenter placed a laptop on the edge of a table and told the female experimenter, “I've got that report of yours on my laptop. Remind me about it at the end.” Participants donned a body motion tracking shirt and hat and were calibrated before being seated at a computer to take part in a math test. The test consisted of two stages of 30 multiplication questions with three multiple choices. Pilot testing indicated people scored ~75% correct.

After the first stage, the male experimenter excused himself while the female experimenter explained what the second stage would entail. She told them what we had found and hoped to continue to find was that standing improves math ability, purposely violating good experimental practice to give the impression that it was normative to perform well on the second stage. In addition, participants were offered £5 if they performed better. They were also told that since they were standing they would be unable to reach the keyboard, so it was also their task to mentally keep track of approximately how many they calculated correctly, but not to voice this. That is, they were encouraged to claim they performed better on the second stage and they were aware there was no way to verify their claim. At this point the female experimenter accidentally knocked the laptop to the floor. She quickly expressed relief saying, “Thank God the cameras were off,” implying that only she and the participant were witnesses to the accident.

The second block was initiated as the male experimenter re-entered the room. The block was designed to become increasingly difficult over time, such that the absolute difference between the three multiple choices was smaller on all trials in comparison to the first stage and that the time to respond was gradually reduced with each successive trial. All participants in a norming test performed worse on the second stage.

After completing the math test, participants were asked a baseline question (“Did you feel the second stage took more or less time to complete?”) and a critical question (“Did you feel you performed better on the first or the second test?”). The responses to these two questions, from the onset of their reply, constitute the neutral and critical recording periods for the math test. Participants who claimed to have performed better were paid the additional £5. Participants were then thanked for taking part and asked to remain in the kit while the male experimenter took a backup of the data onto his laptop. During this time, the neutral (“Did the math experiment run ok?”) and critical laptop-accident questions (“My computer doesn't seem to be working. Did you see anything happen?”) were posed to the participant and recorded.

### Capturing movement

**Figure 4:**
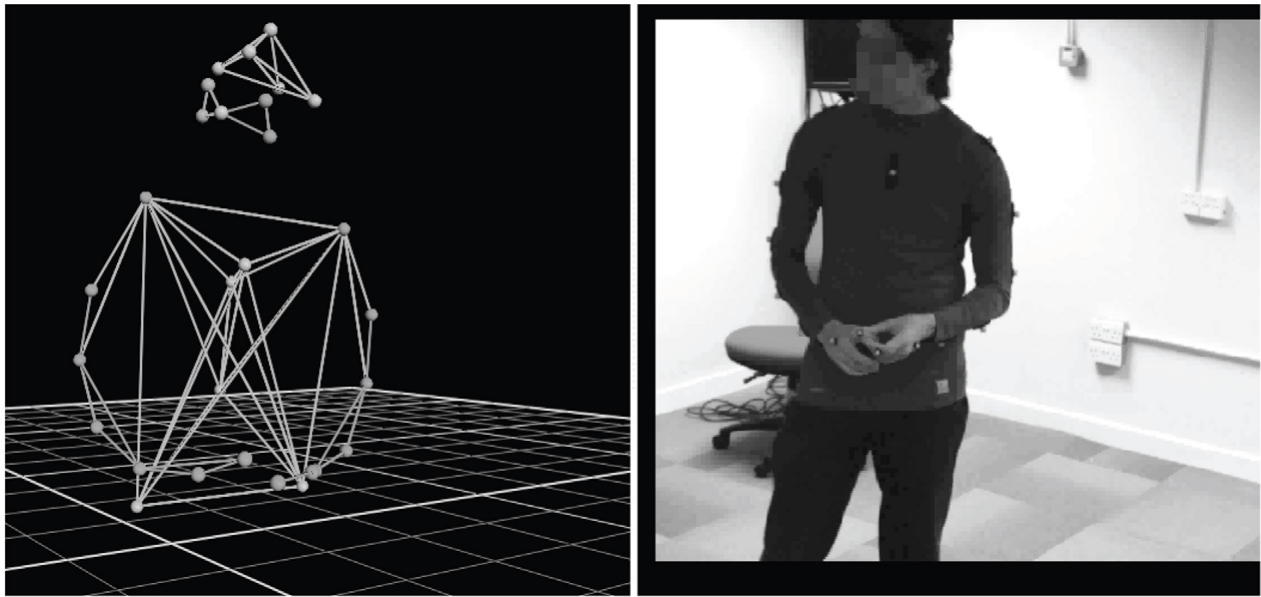
**Marker placement for body, head, and face, reconstructed with an accuracy of 0.1 mm using Vicon Nexus motion tracking software**.

A Vicon Nexus body motion tracker captured three-dimensional movement at 200 Hz by recording near-infrared reflections from 20 plastic markers attached to a tight-fitting shirt and cap. An additional nine markers were attached around the face, on the back of each hand and on the tips of each index finger. Marker positions were captured with an accuracy of 0.1 mm in terms of position in space (Figure 4).

### Movement displacement

We focus here on undifferentiated movements of the arms, head, and upper face. These regions have been targeted in deception research as being especially relevant for detection purposes (Ekman and Friesen, 1969, 1972; Vrij et al., 1996, 1997; Hill and Craig, 2002; DePaulo et al., 2003; Jensen et al., 2010; Hurley and Frank, 2011). In the majority of these previous studies, participants are asked to rate the frequency, duration, or functional purpose of the movements, such as whether the movement has communicative intent (e.g., gestures used to emphasize verbal statements) or is unintentional (e.g., a “leakage” cue flashed across the face). In the current work, we avoid the assumptions needed to make these distinctions, evaluating only the rhythmic sequences of movement over time.

As mentioned, the output of the motion tracker system is in three-dimensional coordinate positions across multiple body markers; and as such, we need to convert position to a single-dimensional measure of movement displacement. To begin, we first averaged the three-dimensional coordinate positions of body markers within each region of interest. For the arms, this includes six points distributed across right/left forearms, hands, and wrists; for the head, five points distributed across the top, right/left, and back/front; and for the face, five points distributed across the eyes and nose, thus minimizing influences from speech articulation.

**Figure 5:**
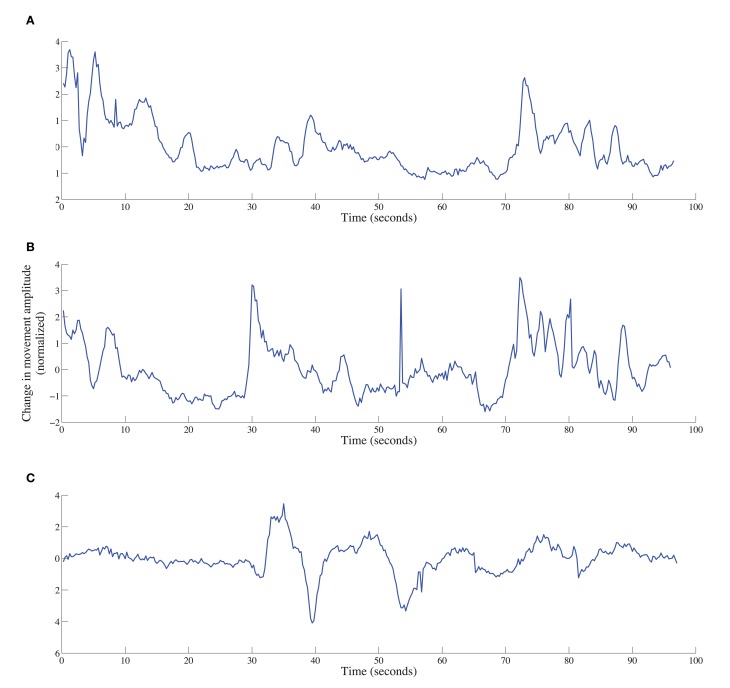
**Time series of movement displacement (based on Euclidean distance) for arms (A), head (B), and upper face (C) for a deceptive responder in the math-test condition**.

Averaging produces a single vector of coordinate positions for each region. Change in movement displacement was computed over windows of 250 ms, equivalent to 20 time steps (based on a sampling rate of 200 Hz). For arms and head, this was done by averaging the Euclidean distances between contiguous (x, y, z) coordinate positions in the moving window. A sample time series is shown in Figure 5. For the face, a slight modification was made based on the observation that movements of the face will co-vary with movements of the head. To remove this influence, Euclidean distances were computed between each face point and a composite head position, and then averaged in the moving window of 20 time steps.

### Parameter selection

The generated displacement time series were normalized (mean zero and standard deviation of one) and used for the RQA and MSE analyses. It should be noted that although the movements here differ from those typically used in the motor control literature, they are still amenable to non-linear analyses and interpretation. Various types of movements have been assessed using a similar approach; for example, changes in the angular velocity of hand movements (Stephen et al., 2009), and movement displacement in the video recordings of facial/head movements (D'Mello, 2011). The main requirement for these analyses is a movement signal that is thought to be generated by a complex system. However, the parameters for RQA and MSE still need to be uniquely specified for signal source in order to avoid spurious or unaccounted structure.

For RQA, the critical parameters correspond to time delay, embedding dimension, and radius for determining whether two points in phase space are sufficiently close (with radius expressed as a percentage of the standard deviation of a normalized time series). Following Shockley (2005) and Shockley et al. (2003), we selected parameter values by first conducting RQA on four randomly selected time series across multiple embedding dimensions, along a range of delay and radius parameter values. Using a surface plot, we plotted the recurrence rate (y-axis) from each analysis, for each embedding dimension, as a function of delay (x-axis) and radius (z-axis). This produces multiple three-dimensional landscapes of valleys and peaks corresponding to recurrence rates that rise or fall depending on parameter value combinations. The optimal parameters are those that are in the flat regions of each series landscape, thus ensuring that the values are stable and not reflecting idiosyncratic change (i.e., small increases or decreases in the selected embedding dimension, time delay, and radius would have little effect on recurrence rates). It is also typical to select values that produce an overall recurrence percentage around 5% and that avoid ceiling effects in determinism. As such, we settled on an embedding dimension of three, a delay of eight, and radius of 15% for all analyses^8^.

For MSE, parameter selection is more straightforward. Here, we followed the precedent of Costa et al. (2005) in setting the parameters corresponding to sample entropy and coarse-graining. As described in the previous section, we began with two-point sequences that were extended by a third point. We also used a threshold radius of 15%, which like RQA, sets the boundary of whether time points are considered similar, and is expressed as a percentage of time series standard deviation. Coarse-grained versions of the original series, in which sample entropy was computed, were reduced by a factor of 2–9 (retaining the original series with a factor of one). This is depicted in Figure 3,^9^.

### Participants

Data from 28 participants were analyzed in this study (18 females and 10 males, mean age 22.5 years old). Most participants were consistent in how they responded between the math-test and laptop-accident conditions, either lying in both or telling the truth in both. However, six participants split their responses between conditions, telling a lie in one and the truth in another. Also, due to some data loss with the Vicon motion tracking system, movements for six participants were unavailable in the accident condition and unavailable for one participant in the math-test condition. In the end, for all analyses, there were 26 deceptive time series (combined across the math-test and laptop-accident conditions; 16 participants; 3 males and 13 females), and 21 truthful time series (combined across the math-test and laptop-accident conditions, 17 participants; 5 males and 12 females).

### Data preparation

Responses in the math-test and laptop-accident conditions were combined for all analyses. This combination was done partly for purposes of generalizability, as the structure of movements associated with deception should be somewhat consistent across similar contexts, thus bolstering claims of detectability. The other reason is more pragmatic, as limitations in statistical power for the RQA and MSE analyses warranted combination. This is often a consequence of using previously collected datasets, particularly sets that involve naturalistic, and somewhat noisy, expressions of behavior. As such, our claims are somewhat limited (an issue we address in the Discussion), but nevertheless, the goals of introducing non-linear measures to the deception literature and relating these measures to the underlying cognitive processes involved in deception are still intact. It should be noted, however, that the pattern of results presented here in fact holds in each case of deception separately.

### Statistical approach

For the displacement and RQA determinism results, differences between deception and truth, across neutral and critical questions, were analyzed using linear mixed effects models. Given that participants sometimes contributed to both or only one of the deceptive responses across conditions, participant and condition variables were entered as random factors in the model to control for associated random variance. Also, because the error term in this model class is not amenable to traditional *F*-test methods for computing a *p*-statistic, an MCMC method was instead used for estimating statistical significance (see Pinheiro and Bates, 2000; Baayen et al., 2008). Next, for MSE curves, differences between relevant groups were analyzed by generating intercept and slope coefficients for each participant's time series data, using a curve-fitting model with linear fit. The resulting coefficient terms were then compared across deceptive and true responses using a two-sample *t*-test.

## Results and interpretation

In this section, we begin with the results of movement displacement, an aggregate measure of magnitude change that has traditionally been used in analytic approaches that average over time series. We then turn to our two non-linear measures, RQA and MSE, that may be useful in capturing additional information about movement dynamics.

### Displacement results

**Figure 6:**
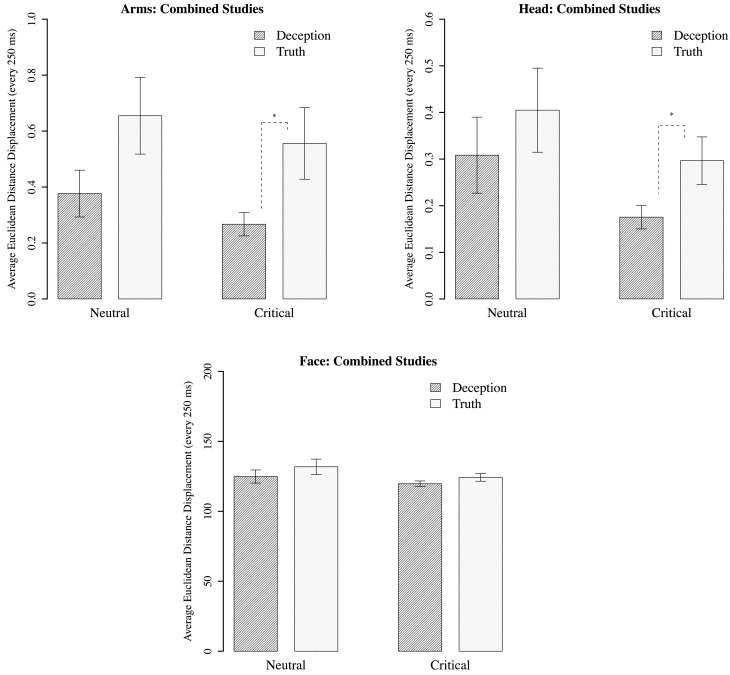
**Mean Euclidean distance displacement (every 250 ms) for motion regions corresponding to the arms, the head, and the upper face (combined for math-test and laptop-accident conditions).** Standard error plotted for each bar. Dark bars are participants who lied during the critical phase; white bars are those who told the truth. Bars are grouped according to neutral question (“Did the math experiment run ok?”), and critical questions (math performance + laptop scenario). ^*^p < 0.05.

Separate analyses were conducted on the arms, head, and upper face regions^10^. In comparing deception with truth, the neutral questions showed no statistically significant differences across all three motion regions. However, for critical questions, the movements of the arms and head reveal significantly less displacement in deception than the truth; for arms, *B* = 0.264, *p* = 0.022; for head, *B* = 0.121, *p* = 0.038. There are no statistically significant differences in displacement for face movements. And for all regions, there were no significant differences between neutral and critical questions for deception or truth (see Figure 6).

For critical questions, we replicated the basic effect found by Eapen et al. (2010), who found less movement for deception across all motion points. Here, using a slightly different operationalization of displacement, decreases were isolated to the arms and head. This finding may suggest that participants are seeking to minimize incriminating behaviors by clamping down on their movements. Conversely, the null finding for the face suggests that the generated movements are much more subtle and spontaneous, and the same control exhibited over the arms and head is not possible. But this may be because the wrong level of movement has been examined, leaving open the possibility that non-linear measures offer a more sensitive means of identifying differences between conditions.

Another issue that is evident from Figure 6 is the lack of significant differences between the neutral and critical questions. Yet the direction of mean values for neutral questions is very similar to that of the critical. Given that the neutral questions always preceded the critical in the experimental setup, participants who cheated on the math test or who were witnesses to the experimenter dropping a computer, may anticipate that a follow-up question will be asked that requires deception (such as being asked about their performance or why the computer was broken). Thus, their response behavior during the neutral question may indicate a preparation to lie that is ultimately expressed when a deceptive response is required. Whether the behavioral system was poised to react in this way is difficult to interpret from movement magnitude alone. Again, non-linear measures may prove useful in clarifying this issue.

### Recurrence quantification analysis results

**Figure 7:**
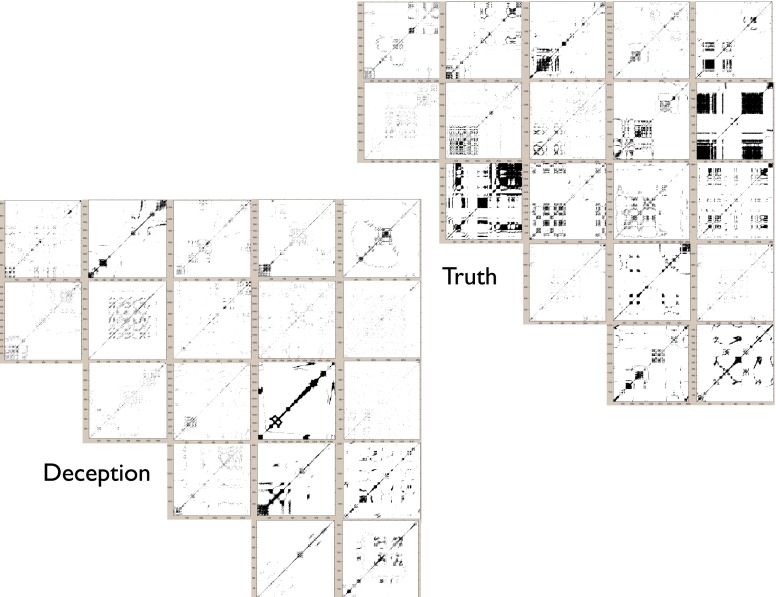
**For upper face movements, mosaic of recurrence plots for randomly selected subset of deceptive and truthful responses for critical questions.** Deception is shown in the lower panel and truth in the upper panel. For truth, there is overall higher determinism than deception, as indicated by the greater percentage of recurrent diagonal lines. Each plot shown in this array is a reflection of the “recurrences” of face movements over time; the more points there are, the more the time series of movements exhibits similar fluctuations. Glancing at the plots does reveal that Truth plots seems to have more dense appearance of recurrence structures (for details on method, see Figure 1). This is quantified using the Determinism percentage shown in Figure 8.

**Figure 8:**
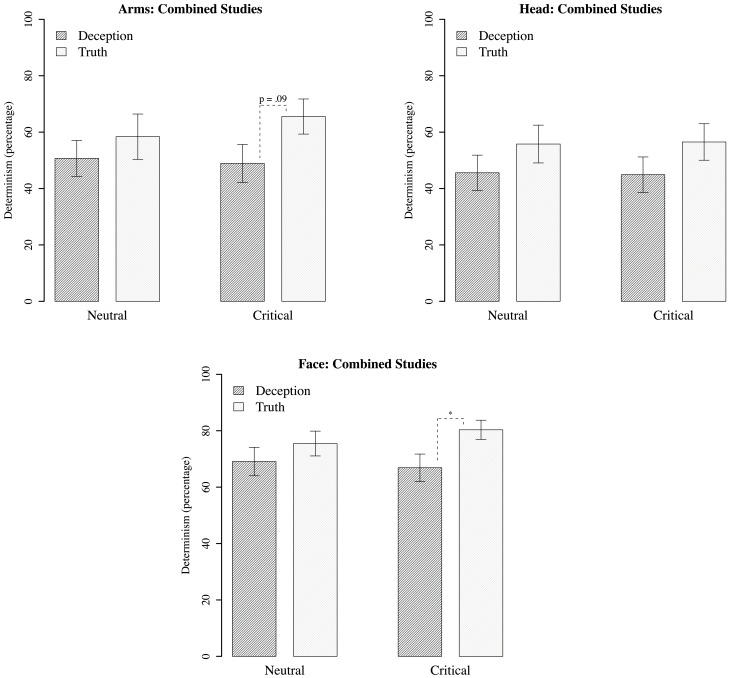
**Mean percentage of determinism for RQA. Standard error plotted for each bar.** ^*^p < 0.05.

For each motion region of interest, measures of percentage recurrence, and determinism were generated based on recurrence plots for deceptive and true responses (Figure 7). The recurrence rate for all analyses were within 4–8%, and did not differ between comparisons of deception vs. truth, or neutral vs. critical questions. However, determinism rate did show statistically significant differences between groups, most notably in upper face movements, with less determinism in deception than in the truth, *B* = 0.126, *p* < 0.05 (Figure 8). There was also marginally less determinism in deception with arm movements, *B* = 0.135, *p* = 0.09; but for head movements, no statistically significant differences were found. There were also no significant differences within neutral questions, and in comparison with the critical questions.

The trend for all regions is for less determinism for the critical questions during deception. This is most safely concluded for the upper face, with some cautious support for arm movements. Even so, this is suggestive that stability, as assessed by determinism, decreases in deception. Although it may be tempting to draw the conclusion that less movement causes a drop in determinism, the results of the upper face indicate otherwise, as no differences were found with displacement (based on the previous analysis). In other words, movement displacement appears to be independent of the influences driving determinism. That is, the non-linear dynamics of the motion reveals new detail about the act of deception that is unavailable to the oft-used frequency counts of more or less movement in prior research.

As with displacement, the pattern of determinism between deceptive and truthful responses was also similar for neutral and critical questions. That is, there were lowered levels of determinism when participants both anticipated and expressed a lie. However, although there is decreased determinism/stability, it is not necessarily characterized by meaningful complexity. Before considering what a decrease in stability might mean in a deceptive context, we interpret the results alongside the MSE analysis.

### Multiscale entropy analysis

**Figure 9:**
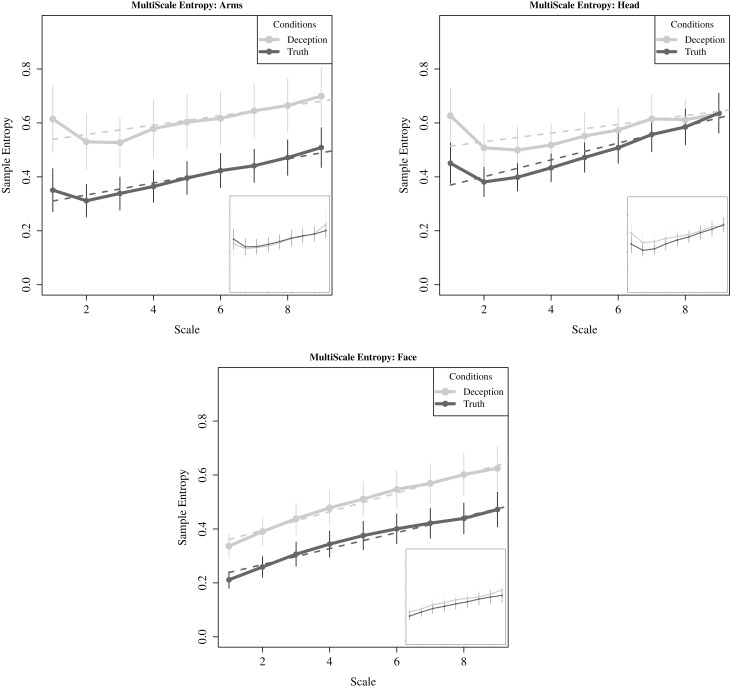
**For critical questions, sample entropy plotted across increasing scale lengths, i.e., lower frequencies (solid lines).** Curve fitting to individual participant data was conducted using linear fit models for the three motion regions. The average intercept and slope shown here (dashed lines). Points represent mean values of sample entropy for each region, with standard error also plotted. The inset plots in each subfigure correspond to movements generated while responding to the neutral question. There are no significant differences between conditions.

As a reminder, MSE relies on sample entropy, a measure that evaluates the repetition of consecutive sequences in a time series (as opposed to variance). Sample entropy is then plotted over multiple time scales increasing in length, with time scales derived from the original movement time series. For each deceptive and truthful response, within each motion region, an MSE curve is generated and fitted with a linear model. To compare the relative complexity between groups, the resulting intercept coefficients for deceptive and truthful responses are evaluated using two-sample *t*-tests. In this way, differences across all scales can be evaluated in one statistic. The slope terms are also examined to compare differences in the rate by which complexity increases over scales. Composite slopes are shown in Figure 9.

For the intercept coefficients, we found statistically significant differences with the movements of the upper face, *t*_(41)_ = 1.976, *p* < 0.05; and once again marginal statistical significance for the arms, *t*_(44)_ = 1.654, *p* = 0.09. There are no statistically significant differences for the head. Thus, the pattern for the upper face and the arms is for greater relative complexity with deception compared to the truth. Next, turning to the rate in which complexity increases for both deception and truth, there is equivalent gain for all regions except the head, where the complexity in the truth rises at a faster rate than deception, *t*_(42)_ = 2.27, *p* < 0.05. Here, truth and deception converge at the larger timescales, and may account for the failure in finding significant differences between deception and truth. Finally, for neutral questions, complexity was present in the neutral responses, but as has been evident in the previous analyses, there were no differences with critical questions.

**Figure 10:**
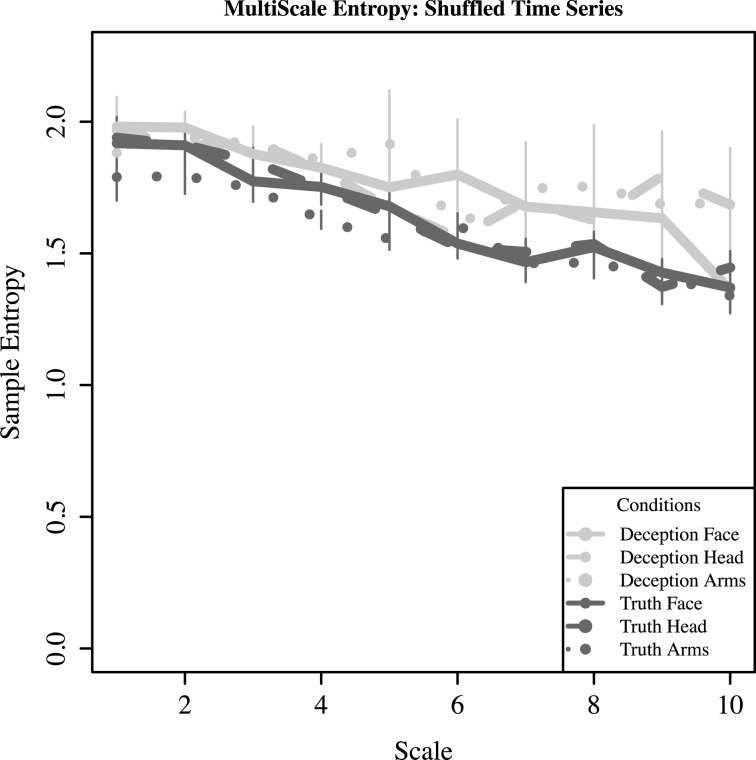
**For shuffled time series (randomized across bins of 2000 ms), mean sample entropy and standard error is plotted across increasing scale lengths (1–10)**.

The findings of greater complexity in deception for the upper face (and somewhat for the arms), is further qualified when one examines what happens when the time series for each response is randomly shuffled while preserving local temporal interdependencies. Binned sequences of 2000 ms sequences were randomly shuffled, effectively removing the time-dependent complexity hypothesized to be present in each series. Based on Figure 10, the monotonic downward slope indicates that the number of new structures drops as the length of the window for coarse-graining increases; thus, there is no new information to be found.

## Discussion

Despite a long tradition in seeking out bodily cues of deception, temporal dependencies in how movement is organized across time have largely been overlooked. In the current paper, we captured these dependencies as emergent properties of a complex system, characterized by structural properties of stability and complexity. Using two non-linear measures, RQA and MSE, we found that the movements about the upper face, and somewhat in the arms, tend to have lower determinism/stability (based on RQA) and higher complexity (based on MSE). These patterns suggest greater flexibility in movement responsiveness that would have remained hidden with a measure of movement displacement alone, as deceptive and truthful facial movements were shown to have similar summary statistics (mean and standard error). Though suggestive, it is important to note that these results are indeed statistically subtle, based on a convenience sample, and also show that the neutral and critical contexts are about the same in most measures within each subject. However, if we take these results for granted, here we consider some potential theoretical implications of these dynamical methods.

These results challenge the notion that the demands introduced by deception exclusively deplete attentional resources and negatively affect the control of movement. That is, rather than only a breakdown in processing, the dynamic signatures of movement are structured in such a way to permit rapid adjustments to emerging demands unique to deceptive, social contexts. To support this claim, we have drawn from a dynamical systems framework for understanding how non-linear systems come to exhibit structured behavior. Human motor behavior is often held up as a primary example, in that patterns of movement are rapidly formed, maintained, and transformed by the release or restriction of system-wide degrees of freedom (Turvey, 1990, 2007; Newell, 1998). What results is increased complexity that speaks to the ability of the motor system to flexibly adjust and adapt to ever-changing situational demands, much like the behaviors of a skilled athlete or a child mastering the ability walk. Such behavior may be necessary in handling the challenges inherent to deception.

Greater flexibility also appears to be present during the neutral questions prior to the actual deception. This finding may point to participants who anticipate that they will need to lie. Although they did not know that they would be put on the spot about their own guilty behaviors (assuming they cheated on the math test), or the guilty actions of another (witnessing a confederate drop a laptop), the possibility of investigative questioning by the experimenter, as well as the experimenter's possible suspicion, was always present. Such a situation would support an increased need for heightened responsiveness (i.e., adaptiveness, see Eapen et al., 2010). One reviewer remarked that this may instead be a sign of a sluggish system that is incapable of rapidly adapting to a more local context. Holding up the results from another perspective, this is a viable interpretation. But one timescale's sluggishness may be another timescale's adaptiveness. The way in which the dynamic signatures seem to be present (i.e., in both neutral and critical questions) suggests adaptiveness at a longer timescale; while this adaptiveness may force more local moments to be under the control of these longer timescales. In other words, the system could be adapting for a future potential event; and before it happens the situation at hand is subject to this structure.

It is also revealing that responsiveness was most apparent in the subtle movements of the upper face. The face has largely been implicated as a “dynamic canvas” for expressive behavior, where intentional and unintentional information about mental states are optimally conveyed (DePaulo, 1992; Rozin and Cohen, 2003). Given that accurate assessments of these states are easily and rapidly seized upon by outside observers (Ambady et al., 2000), it is sensible to hypothesize that these movements need to be particularly flexible in deceptive contexts. Also, unlike the movements of the body and head, the control of the musculature around the eyes may also produce a signal that is most appropriate for the non-linear analyses employed here. Both factors may explain why the reported results were statistically significant for the face alone.

The rapid and small-scale movements in the face are also thought to be susceptible to the inadvertent “leakage” of hidden emotional states (Hill and Craig, 2002; Ekman and Friesen, 2003). Such leakage forms the basis for the *inhibition hypothesis*, whereby attempts to conceal true emotions are revealed in “micro-expressions” of the face that last only tenths of a second (Ekman, 1992; Ekman and Friesen, 2003). Of the few empirical studies that directly examine this claim, evidence suggests that masked negative emotions may elicit the greatest leakage; and that transitory patterns of emotional states, particularly from negative to positive emotions, may also be a predictor of deception (Porter and ten Brinke, 2008; ten Brinke et al., 2011). For the current study, this raises the interesting possibility that the transitional nature of momentary emotional states can account for the current results. However, such transitions are much too coarse-grained to drive the moment-by-moment millisecond fluctuations that were analyzed. Also, given the short duration of participants' interactions with the experimenter, a wide array of changing emotional states is unlikely. Nevertheless, the role of emotions in the current study cannot be discounted. The need to adapt emotional displays to changing circumstances may very well contribute to the increased movement complexity found during deception. Such questions pave a way for future work.

We were limited by certain characteristics of the data, such as participants that unevenly self-selected into deceptive and truthful response groups, and who sometimes lied in both or only one of the math-test and laptop-accident conditions. Statistical power concerns were also limiting, and required us to combine the math-test and laptop-accident conditions. There is also the inescapable fact that statistical effects were somewhat weak. Nevertheless, the upside of the current dataset is that we could draw conclusions from behavior that possesses defining characteristics of deception; that is, participants who deliberately attempted to mislead unsuspecting recipients (a rarity in laboratory-based studies). The dataset also allowed us to examine continuously sampled movements as fluctuations over time. Such data are quite rare in the deception literature, with the exception of a promising line of research that extracts continuous body movements from video recordings (Meservy et al., 2005; Jensen et al., 2010). Although this research uses participants who were instructed to lie and analyses were based on movement displacement alone, a number of these variables have proved to be highly effective in detecting deception. When entered into machine learning models, the classification algorithms produced surprisingly high accuracy rates. Given that we show dynamical measures provide information above and beyond movement displacement, these additional variables could further improve the accuracy of classification.

Lastly, the current approach addresses an important debate in the deception literature concerning the tendency for deceivers to move less. It is unclear whether fewer movements are caused by excessive strategic management to the point that deceivers ironically overcompensate (DePaulo et al., 1988; see also Wegner, 2009) or a strategic move to prevent leakage cues (Burgoon, 2005). This is an important distinction for the lie detector. After all, if the behavior is strategic then its diagnosticity cannot be relied upon. An important facet of accurate lie detection, then, is not only discovering those behaviors that give liars away, but also determining if those behaviors are strategic in an attempt to minimize irrepressible “tells.” Accordingly, dynamical measures of stability and complexity might have a great deal of relevance here. Although people may strategically minimize the overall magnitude of their movements, the dynamical structure of these movements are certainly outside of conscious control. And where a minimization of movement might be considered unintentional, it does not necessarily have to reflect impairment on part of the cognitive system. According to a main hypothesis, when the dynamical properties of movements are examined, what may be expressed are complex patterns of adaptation that emerge in task-specific ways. There are new and exciting ways to spot a liar.

### Conflict of interest statement

The authors declare that the research was conducted in the absence of any commercial or financial relationships that could be construed as a potential conflict of interest.

## Footnotes

^1^This study was originally published as a proceeding article for the Cognitive Science Society. Face data results were not included in the original report.

^2^For a more technical treatment of each approach, we recommend Riley and Van Orden (2005), Dale et al. (2011), and Marwan et al. (2007) for RQA, and Costa et al. (2005) for MSE.

^3^See the excellent resource http://www.recurrence-plot.tk/rqa.php by Norbert Marwan for these and other quantifications.

^4^RQA also produces 11 additional measures that capture further dynamical properties of the recurrence plots, such as averaged diagonal length and length of the longest diagonal line. These measures may provide new directions for analysis, but for current purposes of examining general stability, we focus on a parsimonious set of variables.

^5^It should be noted that RQA also produces an entropy measure based on recurrence plots. This measure is derived from the number of diagonal lines of different lengths, with a greater number indicating greater entropy. However, results can sometimes be difficult to interpret if long diagonal lines are present with many smaller lines. Such a system would be considered highly entropic, yet the presence of long diagonals indicates high stability. The MSE measure allows for a more straightforward interpretation of entropy and complexity. Furthermore, by turning to a measure outside of RQA, we can ensure that the observed patterns are not limited to the RQA-based analysis.

^6^This experiment was conducted under the permission of the UCL Research Ethics Committee.

^7^A reviewer raised the interesting point that had we used different gender roles, our results would have been quite different, citing Wraga et al. (2006) as support. Although this is an intriguing possibility, our aim was to set up a social situation that draws upon social norms about lying and honesty, and correct behavior between participants and experimenter. The goal was to rely upon these schemas of social interaction to elicit a higher rate of spontaneous deception. Had we used other gender roles in doing so, we might expect the rates of deception to decrease. Nevertheless, we believe that the roles used here adhere to reasonable expectations about social interaction and are optimized for the current research question.

^8^The “max norm” method was also used to compute distance between vectors in the reconstructed phase space (Marwan, 2003). Shockley (2005) offers an excellent summary of these issues, and is available as an open access chapter online here: www.nsf.gov/sbe/bcs/pac/nmbs/chap4.pdf

^9^In general, the setting of these specific parameters does not adversely affect the general pattern of results, which hold across a range of these values.

^10^For these and subsequent analyses, the total N for each comparison varied slightly between body regions due to dropped recordings with the Vicon motion tracking system. For arms, there were 26 deceptive and 20 truth time series; for head, there were 23 deceptive and 21 truth time series; and for face, there were 25 deceptive and 20 truth time series.
